# Reply to Kim et al. Acute Hemorrhagic Encephalomyelitis in the Context of MOG Antibody-Associated Disease. Comment on “Chen et al. Rapid Progressive Fatal Acute Hemorrhagic Encephalomyelitis. *Diagnostics* 2023, *13*, 2481”

**DOI:** 10.3390/diagnostics13233549

**Published:** 2023-11-28

**Authors:** Hung-Chieh Chen, Ting-Bin Chen

**Affiliations:** 1 Department of Radiology, Division of Neuroradiology, Taichung Veterans General Hospital, Taichung 40705, Taiwan; hungchiehchen@gmail.com; 2 School of Medicine, National Yang-Ming Chiao Tung University, Taipei 112304, Taiwan; 3 Department of Neurology, Neurological Institute, Taichung Veterans General Hospital, Taichung 40705, Taiwan; 4 Dementia and Parkinson’s Disease Integrated Center, Taichung Veterans General Hospital, Taichung 40705, Taiwan; 5 Center for Geriatrics and Gerontology, Taichung Veterans General Hospital, Taichung 40705, Taiwan

We want to express our appreciation for the insightful comments [1] that you made on our recent article, which discussed a case of acute hemorrhagic encephalomyelitis (AHEM) in a middle-aged male patient. Your analysis provided a valuable perspective on this complex clinical scenario.

Your suggestion that AHEM might be linked to myelin oligodendrocyte glycoprotein antibody-associated disease (MOGAD) is intriguing. We also acknowledge your observation that certain clinical features of AHEM overlap with those of MOGAD, and the similarities between the brain MRI findings [1] are undeniably thought-provoking. We agree that this observation warrants further investigation.

Regarding MOG antibody testing, to clarify, we conducted this test, and the results were negative in our patient. We apologize for any confusion that arose from the omission of this critical detail from our original article. Serum and/or cerebrospinal fluid (CSF) autoantibody tests were performed using a commercial cell-based indirect immunofluorescence assay (Uni Pharma Co., Ltd., Taipei, Taiwan). CSF and serum samples were tested for the presence of autoantibodies against NMDAR, AMPAR1, AMPAR2, CASPR2, LGI1, and GABAbR; the serum sample was also examined for the presence of autoantibodies against AQP4 and MOG. Both CSF and serum tested negative for all the above-mentioned antibodies.

In conclusion, we appreciate your comments and commitment to advancing our understanding of AHEM. Your insights will undoubtedly influence the direction of our future research. We hope that ongoing studies will further outline the intricate relationship between AHEM and MOGAD.

Once again, thank you once for your engagement with our work and enriching the discussion.

## References

[1] Kim S., Eun M.-Y., Lee J.-J., Seok H.Y. (2023). Acute Hemorrhagic Encephalomyelitis in the Context of MOG Antibody-Associated Disease. Comment on Chen et al. Rapid Progressive Fatal Acute Hemorrhagic Encephalomyelitis. *Diagnostics* 2023, *13*, 2481. Diagnostics.

